# Transcriptomic profiles of tissues from rats treated with anticancer drug combinations

**DOI:** 10.1038/sdata.2018.306

**Published:** 2019-01-08

**Authors:** Myrtle Davis, Elaine Knight, Sandy R. Eldridge, Jianying Li, Pierre R. Bushel

**Affiliations:** 1 Toxicology and Pharmacology Branch, Division of Cancer Treatment and Diagnosis, National Cancer Institute, Bethesda, MD, USA; 2 Kelly Government Solutions, Research Triangle Park, NC, USA; 3 Integrative Bioinformatics Group, National Institute of Environmental Health Sciences, Research Triangle Park, NC, USA; 4 Microarray and Genome Informatics Group, National Institute of Environmental Health Sciences, Research Triangle Park, NC, USA; 5 Biostatistics and Computational Biology Branch, Division of Intramural Research, National Institute of Environmental Health Sciences, Research Triangle Park, NC, USA

**Keywords:** Microarrays, Microarray analysis

## Abstract

To achieve therapeutic goals, many cancer chemotherapeutics are used at doses close to their maximally tolerated doses. Thus, it may be expected that when therapies are combined at therapeutic doses, toxicity profiles may change. In many ways, prediction of synergistic toxicities for drug combinations is similar to predicting synergistic efficacy, and is dependent upon building hypotheses from molecular mechanisms of drug toxicity. The key objective of this initiative was to generate and make publicly available key high-content data sets for mechanistic hypothesis generation as it pertains to a unique toxicity profile of a drug pair for several anticancer drug combinations. The expectation is that tissue-based genomic information that are derived from target tissues will also facilitate the generation and testing of mechanistic hypotheses. The view is that availability of these data sets for bioinformaticians and other scientists will contribute to analysis of these data and evaluation of the approach.

## Background & Summary

It is hypothesized that unique patterns of gene expression changes will be associated with drug combinations that have a higher risk of synergistic or additive toxicity as compared with either agent used alone. If this hypothesis is true, these patterns can then be used to provide some initial degree of discrimination between drug combinations with higher risk of combination toxicity. Initial evidence of this has been published where we show that the administration of oxaliplatin in combination with topotecan, or either drug alone, in the rat elicits gene expression changes in the bone marrow that are dependent on the order of the administration and indicative of enhance toxicity^1^.

The drug combinations included in this evaluation were chosen based on common target organ toxicities (that can be replicated in a preclinical model) and the potential for actual clinical use as a combination. The test compounds were administered to a single preclinical species for this proof of concept. Rats were chosen as the test species because they are commonly used in preclinical safety assessment. It is important to note that this initiative focused on gene expression profiles of select tissues and histopathology of those tissues.

Each study in the data set used a combination two of drugs (Table 1). Temsirolimus is an intravenous drug for treatment of renal cell carcinoma^2^. It is a specific inhibitor of mTOR and interferes with synthesis of proteins that regulate proliferation, growth, and survival of tumor cells. Oxaliplatin, a platinum compound, is used to treat colorectal cancer^3^. Its cytotoxicity is thought to result from inhibition of DNA synthesis. Oxaliplatin forms both inter- and intra-strand crosslinks in DNA which prevent DNA replication and transcription, hence causing cell death^4^. Gemcitabine^3^ is used to treat a variety of cancers, particularly breast cancer, ovarian cancer, non-small cell lung cancer, pancreatic cancer, and bladder cancer. It exerts its effect by blocking DNA synthesis resulting in cell death. Sunitinib, sorafenib and erlotinib are inhibitors of several tyrosine kinases and are used for the treatment of primary kidney cancer or imatinib-resistant gastrointestinal stromal tumor in the case of the former drug and non-small cell lung cancer, pancreatic cancer and several other types of cancer in the case of the latter drug^2^. Table 2 lists published gene expression studies (mRNA and miRNA) in the rat and mouse in which the animals were exposed to the same chemicals as in this study either alone or in combination with another drug or chemical.

**Figure 1:**
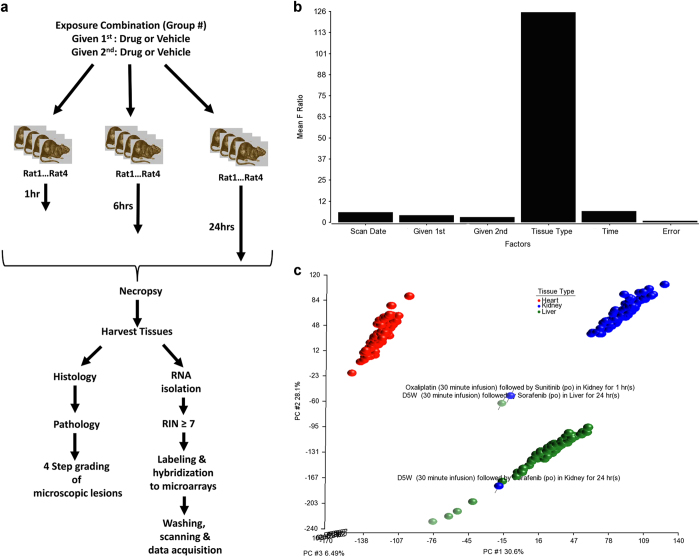
Study design, analysis workflow, sources of variability and principal component analysis. (**a**) Exposure, sample collection and data acquisition workflow. (**b**) Sources of variability for Study 2. The mean F ratio statistics from an ANOVA model of study 2 data is plotted for each factor. (**c**) PCA scatter plot of samples from Study 2.

The study designs are shown in Tables 3, 4, 5. As shown in Fig. 1a, rats were euthanized, necropsied and then tissues and samples prepared for histology and RNA extraction at the times shown. Histopathology was performed on tissue sections to grade microscopic lesions as minimal, mild, moderate or marked. RNA samples were labeled, hybridized to Affymetrix rat microarrays, washed, scanned and data acquired. Quality of the microarray data was confirmed using a variety of approaches (Table 6) and the data normalized by RMA^5,6^.

Herein, we provide a comprehensive gene expression data set from rats exposed to a variety of cancer therapeutics in combinations. We are sharing these data to enable evaluation and testing of unique bioinformatic approaches for analysis of the data.

## Methods

### Animals and Treatments

The 96 male Sprague Dawley rats designated for use in each study were selected from 104 males received from Charles River Laboratories, Inc. (Raleigh, NC). Animals were given a unique identification number for this study by ear punch. The Sprague Dawley rat is an accepted species and strain that is commonly used in preclinical pharmacological and toxicological evaluations of drugs used or intended for use in humans. On Day 1 of the study, the rats were:

- approximately 7–8 weeks of age and weighed between 197.6–274.4 grams in the Temsirolimus and Sorafenib or Sunitinib study.
- approximately 8 weeks of age and weighed between 251.5–336.2 grams in the Oxaliplatin and Sorafenib or Sunitinib study.
- approximately 8–9 weeks of age and weighed between 256.6–307.3 grams in the Gemcitabine and Sorafenib or Erlotinib study.

Teklad Certified Rodent Diet 2016C (Harlan; Madison, WI) and tap water (Birmingham public water supply) were provided ad libitum to the rats during the prestudy and study periods. Analysis of the Certified Rodent Diet 2016C was conducted by the manufacturer. The results of the feed and water analyses are located in the facility records at Southern Research. The animals were individually housed in solid-bottom polycarbonate cages on stainless steel racks in a room maintained at a:

- temperature of 68–76 °F and a relative humidity of 41–82% in the Temsirolimus and Sorafenib or Sunitinib study.
- temperature of 64–79 °F and a relative humidity of 27–70% in the Oxaliplatin and Sorafenib or Sunitinib study.
- temperature of 70–76 °F and a relative humidity of 25–53% in the Gemcitabine and Sorafenib or Erlotinib study.

Cage size and animal care conformed to the guidelines of the Guide for the Care and Use of Laboratory Animals (National Research Council 2011), the U.S. Department of Agriculture through the Animal Welfare Act (Public Law 99–198), and to the applicable Standard Operating Procedures (SOPs) of Southern Research.

The experimental designs and dose procedures used for the three studies are as follows:

### Study 1: Temsirolimus and Sorafenib or Sunitinib

#### Test Articles

Temsirolimus (80 mg; NSC-683864; NSC/sample: 683864/6; Lot No.: BTM-105; SRI Lot No: M30/L-1; expiration date: September 22, 2015), sorafenib, tosylate salt (2.0 g; NSC-724772; NSC/sample: 724772-F/2; Lot No.: BSF-105; SRI Lot No.: M26/L-1; expiration date: April 24, 2019), and sunitinib, malate salt (160 mg; NSC-736511; NSC/sample: 736511-0/2; Lot No.: BST-104; SRI Lot No.: M31/L-1; expiration date: April 27, 2019) were supplied by the National Cancer Institute (Rockville, MD) and received at Southern Research on October 19, 2010. Each test article was shipped on dry ice and subsequently stored frozen (≤−20 °C) under inert gas.

#### Vehicle/Vehicle Control Articles

The following reagents were used in the preparation of dose formulations:

- Ethyl Alcohol, 200 Proof Absolute Alcohol, ACS/USP Grade (Lot No. KID24C; Pharmco-AAPER; Brookfield, CT; recommended retest date April 2012)
- Pluronic® F-68, solid cell culture tested, insect cell culture tested (Lot No. 020M0029; Sigma Aldrich; St. Louis, MO; retest date March 2014)
- Polyethylene glycol, average M.W. 400 (PEG 400) (Lot No. A0284717; Acros Organics; Fair Lawn, NJ; assigned expiration date: September 23, 2011)
- 1, 2 Propanediol, ACS reagent ≥99.5% (propylene glycol; Lot No. MKBD2071; Sigma Aldrich; St. Louis, MO; assigned expiration date: October 21, 2011)
- Saline Solution 0.9% (Lot No. 090615A3; Nova-Tech, Inc.; Grand Island, NE; expiration date: June 2011)
- Sterile Water for Injection; (Lot No. 090818A2; Vedco, Inc.; St. Joseph, MO; expiration date: August 2011)
- Deionized Water (Southern Research Institute; Birmingham, AL; in-house deionized water system)

All reagents were received and stored at room temperature. In addition, polyethylene glycol was stored under nitrogen, protected from light.

### Vehicle Control Formulation Preparation

#### Temsirolimus Vehicle Control (TEM vehicle)

The temsirolimus vehicle control formulation was prepared in three steps. First, a vehicle stock solution was prepared by mixing 1 volume of absolute ethanol with 1 volume of propylene glycol. For the preparation of an intermediate vehicle stock solution, the vehicle stock solution was diluted 1:4 with diluent (5% Tween 80/5% PEG 400 in sterile water) and then swirled/sonicated until a clear solution was obtained. The intermediate stock solution was diluted 1:82 with sterile saline to yield the temsirolimus vehicle control formulation.

#### 10% Pluronic F68/propylene glycol/PEG 400 (15:42.5:42.5, w:w:w)

A solution containing 10% Pluronic F68 was prepared by dissolving Pluronic F68 in deionized water. The 10% Pluronic F68 solution was added to the appropriate amount of propylene glycol, followed by stirring/sonication, as required. Then the appropriate amount of PEG 400 was added to the 10% Pluronic F68/propylene glycol solution and the mixture was stirred/sonicated until the contents were visibly homogenous. The final solution contained 10% Pluronic F68/propylene glycol/PEG 400 (15:42.5:42.5, w:w:w; F68/PG/PEG400).

### Test Article Formulation Preparation

#### Temsirolimus

Dose formulations of temsirolimus were prepared in three steps. First a stock solution of temsirolimus (20 mg/mL) was prepared by dissolving temsirolimus in absolute alcohol and then mixing with an equal volume of propylene glycol, with sonication until a clear solution was obtained. The stock solution of temsirolimus was prepared once and used for preparation of the dose formulation of temsirolimus prepared on each day of dosing; the stock solution of temsirolimus was stored refrigerated and protected from light between each day of use. An intermediate stock solution (5 mg/mL) of temsirolimus was prepared by mixing, with stirring and sonication, as required, one volume of the temsirolimus stock solution (20 mg/mL) with 3 volumes of diluent (5% Tween 80/5% PEG 400 in sterile water). Each temsirolimus dose formulation was prepared by mixing, with stirring and sonication, as required, 1 volume of the intermediate stock solution (5 mg/mL) with 82 volumes of sterile saline. The nominal concentration of temsirolimus in each dose formulation was 0.06 mg/mL.

#### Sorafenib

Dose formulations of sorafenib were prepared in 10% Pluronic F68:propylene glycol:PEG 400 (15:42.5;42.5; w:w:w) to contain a nominal concentration of sorafenib tosylate of 2.5 mg/mL. For preparation, the required amount of sorafenib tosylate was added to the vehicle and the mixture was stirred/sonicated until a visually uniform solution was obtained.

#### Sunitinib

Dose formulations of sunitinib were prepared in sterile water to contain a nominal concentration of sunitinib malate of 0.5 mg/mL. For preparation, the required amount of sunitinib malate was weighed and mixed with the appropriate amount of sterile water. The formulation was stirred until a clear solution was obtained.

#### Dose Formulation Concentration Analysis

Upon preparation, duplicate samples (0.5 mL) of each dose formulation were obtained and stored frozen (approximately −20 °C) for possible future analysis.

#### Randomization and Group Assignment

Rats were assigned to their respective treatment group using a computer-generated randomization procedure. Body weights required for randomization were obtained during Week -1. After randomization, each of the 96 rats was assigned to one of eight treatment groups as indicated in study 1 experimental design (Table 3).

#### Treatments

Dosing of the study animals was accomplished on two consecutive days, wherein two rats per dose group were dosed on the individual days of dosing. For each rat, the day of dosing was designated as Day 1. Each rat was administered a single bolus intravenous (iv) dose of temsirolimus or appropriate vehicle formulation, as described in the table. Thirty (30) minutes after iv administration of temsirolimus or vehicle formulation, sorafenib, sunitinib, or respective vehicle formulation was administered orally (*per os*; PO). IV doses were administered in a dose volume of 5 mL/kg. PO doses were administered in a dose volume of 10 mL/kg. Dose volumes were based on the most recent individual body weights taken.

### Study 2: Oxaliplatin and Sorafenib or Sunitinib

#### Test Articles

Oxaliplatin (1.2 g; NSC-266046; NSC/sample: 266046-0/7; Lot No.: 100401; SRI Lot No.: M32/L-1; expiration date: unknown), sorafenib, tosylate salt (2.0 g; NSC-724772; NSC/sample: 724772-F/2; Lot No.: BSF-105; SRI Lot No.: M26/L-1; expiration date: April 24, 2019), and sunitinib, malate salt (160 mg; NSC-736511; NSC/sample: 736511-0/2; Lot No.: BST-104; SRI Lot No.: M31/L-1; expiration date: April 27, 2019) were supplied by the National Cancer Institute (Rockville, MD) and received at Southern Research on September 30, 2010 (sorafenib) or October 19, 2010 (oxaliplatin and sunitinib). Each test article was shipped on dry ice. Subsequently, oxaliplatin was stored refrigerated; sorafenib and sunitinib were stored frozen (≤−20 °C) under inert gas.

#### Vehicle/Vehicle Control Articles

The following reagents were used in the preparation of the dose formulations:

- Deionized Water (DI-Water) (Southern Research Institute; Birmingham, AL; in house deionized water system)
- 5% Dextrose Injection, USP (PSS World Medical, Inc.; Kennesaw, GA; Lot No. J0K942; expiration date: November 30, 2011)
- Pluronic® F-68, solid cell culture tested, insect cell culture tested (Sigma-Aldrich; St. Louis, MO; Lot No. 020M0029; retest date: March 2014)
- Polyethylene glycol, average M.W. 400 (PEG 400) (Acros Organics; Fair Lawn, NJ; Grouping No. A0284717; expiration date: September 23, 2011)
- 1, 2 Propanediol, ACS reagent ≥99.5% (propylene glycol) (Aldrich; Milwaukee, WI; Lot No. MKBD1803; expiration date: October 21, 2011)
- Sterile Water for Injection (Vedco Inc.; St. Joseph, MO; Lot No. 090818A2; expiration date: August 2011)

All reagents were received and stored at room temperature. In addition, polyethylene glycol was stored under nitrogen and propylene glycol was stored protected from light.

### Vehicle Control Formulation Preparation

#### 10% Pluronic F68/propylene glycol/PEG 400 (15:42.5:42.5, w:w:w)

A solution containing 10% Pluronic F68 was prepared by dissolving Pluronic F68 in deionized water. The 10% Pluronic F68 solution was added to the appropriate amount of propylene glycol, followed by stirring/sonication, as required. Then the appropriate amount of PEG 400 was added to the 10% Pluronic F68/propylene glycol solution and the mixture was stirred/sonicated until the contents were visibly homogenous. The final solution contained 10% Pluronic F68/propylene glycol/PEG 400 (15:42.5:42.5, w:w:w; F68/PG/PEG400).

### Test Article Formulation Preparation

#### Oxaliplatin

Dose formulations of oxaliplatin were prepared in 5% dextrose in water (D5W) to contain a nominal concentration of oxaliplatin of 3.75 mg/mL. For preparation, the required volume of D5W was added to a weighed amount of oxaliplatin and the mixture was stirred until a visually uniform solution was obtained.

#### Sorafenib

Dose formulations of sorafenib were prepared in 10% Pluronic F68:propylene glycol:PEG 400 (15:42.5:42.5; w:w:w) to contain a nominal concentration of sorafenib tosylate of 2.5 mg/mL. For preparation, the required amount of sorafenib tosylate was added to the vehicle and the mixture was stirred/sonicated until a visually uniform solution was obtained.

#### Sunitinib

Dose formulations of sunitinib were prepared in sterile water to contain a nominal concentration of sunitinib malate of 0.5 mg/mL. For preparation, the required amount of sunitinib malate was weighed and mixed with the appropriate amount of sterile water. The formulation was stirred until a clear solution was obtained.

#### Dose Formulation Concentration Analysis

Upon preparation, duplicate samples (0.5 mL) of each dose formulation were obtained and stored frozen (approximately −20 °C) for possible future analysis.

#### Randomization and Group Assignment

Prior to dosing on Day 1, the catheter of each rat was checked for patency. All patent catheters were flushed with 0.2 to 0.3 mL of 0.9% saline, followed by 0.1 mL of heparin lock solution. Each of the 96 rats with patent catheters was arbitrarily assigned to one of eight treatment groups as indicated in study 2 experimental design (Table 4).

#### Treatments

Dosing of the study animals was accomplished on two separate days. Rats in Groups 1, 3, 5, and 7 were dosed on one day and rats in Groups 2, 4, 6, and 8 were dosed two days later. For each rat, the day of dosing was designated as Day 1.

Each rat was administered the drug by iv infusion over a 30-minute interval, of oxaliplatin or corresponding vehicle formulation (D5W), as described in Table 4. Thirty (30) minutes after iv administration of oxaliplatin or corresponding vehicle formulation, sorafenib, sunitinib, or the respective vehicle formulation was administered PO. IV infusion doses were administered at a flow rate of approximately 4 mL/kg/30 min infusion. Actual infusion rates were based on the mean body weight of animals dosed on each day of dosing. PO doses were administered in a dose volume of 10 mL/kg.

### Study 3: Gemcitabine and Sorafenib or Erlotinib

#### Test Articles

Gemcitabine, hydrochloride salt (500 mg; NSC-613327; NSC/sample: 613327-S/2; Lot No.: ML-10-53; SRI Lot No.: M24/L-1; expiration date: unknown); sorafenib, tosylate salt (2 g; NSC-724772; NSC/sample: 724772-F/2; Lot No.: BSF-105; SRI Lot No.: M26/L-1; expiration date: April 24, 2019), and erlotinib, hydrochloride salt (1.6 g; NSC-718781; NSC/sample: 718781/4; Lot No.: BBE-104; SRI Lot No.: M25/L-1; expiration date: November 19, 2018) were supplied by the National Cancer Institute and received from Fisher BioServices (Rockville, MD; gemcitabine, sorafenib) or SAIC-Fredrick (Frederick, MD; erlotinib) on September 30, 2010.

All three test articles were received on dry ice and subsequently stored frozen at approximately −20 °C. Sorafenib and erlotinib were stored frozen (−20 °C) under inert gas.

#### Vehicle/Vehicle Control Articles

The following reagents were used in the preparation of dose formulations:

- Carboxymethylcellulose sodium salt, medium viscosity (CMC; 400–800 cps; Sigma Aldrich, St. Louis, MO; Lot No.: 010M0089; assigned expiration date: June 22, 2011).
- Deionized water (Southern Research Institute; Birmingham, AL; in-house deionized water system).
- Pluronic® F-68 (solid, cell culture tested, insect cell culture tested; Sigma Aldrich; St. Louis, MO; Lot No.: 020M0029; retest date: March 2014)
- Polyethylene glycol (PEG 400; average M.W. 400; Acros Organics; Fair Lawn, NJ; Lot No.: A0284717; assigned expiration date: September 23, 2011)
- 1, 2-Propanediol, ACS reagent ≥99.5% (propylene glycol; Sigma Aldrich; St. Louis, MO; Lot No.: MKBD1803; assigned expiration date: September 23, 2011)
- Sterile water for injection (Vedco, Inc.; St. Joseph, MO; Lot No.: 090818A2; expiration date: August 2011)
- Saline solution 0.9% (Nova-Tech, Inc.; Grand Island, NE; Lot No.: 090615A3; expiration date: June 2011)

All reagents were received and stored at room temperature. In addition, polyethylene glycol was stored under nitrogen; propylene glycol was stored protected from light.

### Vehicle Control Formulation Preparation

#### 0.5% CMC

A weighed quantity of CMC was slowly added, with stirring, to approximately 90% of the target volume of sterile water. After addition of the total quantity of CMC, the formulation was brought to final volume with sterile water and stirred until a visually uniform solution was obtained.

#### 10% Pluronic F68/propylene glycol/PEG 400 (15:42.5:42.5; w:w:w)

A solution containing 10% Pluronic F68 was prepared by dissolving Pluronic F68 in deionized water. The 10% Pluronic F68 solution was added to the appropriate amount of propylene glycol, followed by stirring/sonication, as required. Then the appropriate amount of PEG 400 was added to the 10% Pluronic F68/propylene glycol solution and the mixture was stirred/sonicated until the contents were visibly homogenous. The final solution contained 10% Pluronic F68/propylene glycol/PEG 400 (15:42.5:42.5; w:w:w).

### Test Article Formulation Preparation

#### Gemcitabine

The dose formulation of gemcitabine was prepared in 0.9% saline to contain a nominal concentration of gemcitabine hydrochloride of 4 mg/mL. For preparation, the required amount of gemcitabine hydrochloride was mixed with saline until dissolution of the gemcitabine hydrochloride occurred.

#### Sorafenib

The dose formulation of sorafenib was prepared in 10% Pluronic F68:propylene glycol:PEG400 (15:42.5;42.5; w:w:w) to contain a nominal concentration of sorafenib tosylate of 2.5 mg/mL. For preparation, the required amount of sorafenib tosylate was added to the vehicle and the mixture was stirred/sonicated until a visually uniform solution was obtained.

#### Erlotinib

The dose formulation of erlotinib was prepared in 0.5% CMC to contain a nominal concentration of erlotinib hydrochloride of 15 mg/mL. For preparation, the required amount of erlotinib hydrochloride was mixed with the appropriate volume of 0.5% CMC. The formulation was stirred, sonicated (approximately 10 min), and homogenized (approximately 2 min) until a uniform suspension was obtained.

#### Dose Formulation Concentration Analysis

Upon preparation, duplicate samples (0.5 mL) of each dose formulation were obtained and stored frozen (approximately −20 °C) for possible future analysis.

#### Randomization and Group Assignment

Rats were assigned to their respective treatment group using a computer-generated randomization procedure. Body weights required for randomization were obtained during Week -1. After randomization, each of the 96 rats was assigned to one of eight treatment groups as indicated in study 3 experimental design (Table 5). On the day of randomization, the hair was clipped from the inguinal area of each animal to facilitate skin collection at necropsy. Re-clipping was accomplished prior to necropsy as required.

#### Treatments

Dosing of the study animals was accomplished on two consecutive days, wherein two rats per dose group were dosed on the individual days of dosing. For each rat, the day of dosing was designated as Day 1. Each rat was administered a single bolus iv dose of gemcitabine or appropriate vehicle formulation as described Table 5. Thirty (30) minutes after iv administration of gemcitabine or vehicle formulation, sorafenib, erlotinib, or vehicle formulation was administered PO. IV doses were administered in a dose volume of 5 mL/kg. PO doses were administered in a dose volume of 10 mL/kg. Dose volumes were based on the most recent individual body weights taken. Dose formulations containing erlotinib were suspensions and these were stirred for at least 5 min prior to use for dosing and continuing throughout their period of use for dosing.

### Clinical Observations

Rats were observed twice daily during the quarantine and study periods for signs of mortality and moribundity. Detailed clinical examinations of each rat were collected prior to euthanasia.

### Body Weights

Each animal was weighed on the day prior to dosing (for the calculation of infusion rate) and on Day 1 prior to dosing.

### Tissue Collections and RNA Isolation

At 1, 6, or 24 hours (h) after the completion of dosing, four (4) rats per dose group were euthanized by CO_2_ asphyxiation. Immediately after euthanasia, selected tissues were collected for RNA isolation and for histopathology. Tissue samples for RNA isolation were collected first and collected as quickly as possible following animal euthanasia.

#### Tissues for RNA Isolation

Sections of each of the following tissues were collected from each rat for RNA isolation:

**Study 1:** Bone marrow (left femur), heart (apex, with left and right ventricles), liver (left lateral and median lobes).

**Study 2:** Kidney (left), heart (apex, with left and right ventricles), liver (left lateral and median lobes).

**Study 3:** Liver (left lateral and median lobes), heart (apex, with left and right ventricles), skin (inguinal area).

Samples of kidney, heart and liver collected for RNA isolation were cut into sections ≤2 mm in thickness and immediately placed into pre-filled tubes containing RNAlater® (Applied Biosystems; Foster City, CA). Bone marrow was flushed from the bone using RNAlater®.

After collection into RNAlater®, tissues were maintained refrigerated (approximately 4-5 °C) for at least 24 h and then stored at or below −20 °C.

#### Tissues for Histopathology

Samples of each of the following tissues were collected for histopathology and were fixed in 10% neutral buffered formalin:

**Study 1:** Bone marrow (right femur), heart, liver.

**Study 2:** Liver, heart, kidney.

**Study 3:** Liver, heart, skin (inguinal area).

### RNA Isolation and Shipment of Samples

RNA isolation from individual tissues was accomplished using an RNeasy microarray kit (QIAGEN Inc.; Valencia, CA). After extraction, the RNA concentration of each sample was determined using a RiboGreen assay. The RNA Integrity Number (RIN)^7^ of each sample was also determined using an Agilent 2100 Bioanalyzer with 2100 Expert Software (Version B.02.06.S1418; Agilent; Santa Clara, CA). Only samples with an RIN of 7 or higher were deemed acceptable for gene expression analysis (Data Citation 1). Each RNA sample was diluted to the required concentration (250 ng/μL) and then stored at or below −70 °C prior to shipment to Expression Analysis (Durham, NC) for microarray analysis.

### Microarray Analysis

RNA samples were converted into labeled target antisense RNA (cRNA) using the Single-Round RNA Amplification and Biotin Labeling System (Enzo Life Sciences, Farmingdale, NY). Microarray analysis was performed as previously described^1^. Briefly, 2.5 μg of total RNA was converted into double stranded cDNA via reverse transcription using an oligo-d(T) primer-adaptor. This cDNA was purified and used as a template for *in vitro* transcription using T7 RNA polymerase and biotinylated ribonucleotides. The resulting cRNA was purified using magnetic beads and quantitated using spectrophotometry. Next, 11 μg of purified cRNA was fragmented using a 5X fragmentation buffer (200 mM Tris-Acetate, pH 8.1, 500 mM KOAc, 150 mM MgOA), then a hybridization cocktail was prepared and added to the fragmentation product using the Hybridization, Wash and Stain kit (Affymetrix, Santa Clara, CA), applied to Affymetrix^TM^ GeneChip® Rat Genome 230 2.0 Arrays, and incubated at 45 °C for 16 h. Following hybridization, arrays were washed and stained using standard Affymetrix procedures before scanning on the Affymetrix GeneChip Scanner 3000 using factory PMT settings to generate array images. Data extraction from the images was completed with the GeneChip Operating Software (GCOS) to generate CEL and CHP files. The microarray analysis method is an expanded version of descriptions in our related work^1^.

Affymetrix raw CEL files were preprocessed using the robust multichip average (RMA) algorithm^5,6^ which includes background correction, quantile normalization, summarization by the median polish approach and log base 2 transformation. The Bioconductor^8^ R packages “affy”, “affyPLM”, “affyQCReport”, “simpleaffy” and “arrayQualityMetrics” were used for array data quality assessment and visualization.

### Code availability

No custom code was used for analysis. Microarray analysis and technical validation were performed using R function calls in the publicly available Bioconductor^8^ R packages.

### Histopathology

#### Histology

All collected tissues from rats in each dose group were processed for histopathological examination. The fixed tissues were trimmed, processed, and microtomed (approximately 5-µm sections). The tissue sections were mounted on glass slides, stained with hematoxylin and eosin, and coverslipped. In Study 1 (Temsirolimus and Sorafenib or Sunitinib) special stains (trichrome and Congo red) were performed on the kidney of one animal (2M14) at the discretion of the pathologist in order to establish a diagnosis.

#### Microscopic Observations

All slides were examined microscopically by a pathologist. Records of gross findings for a specimen from postmortem observations were available to the pathologist when examining that specimen histopathologically. Indication of a histopathology observation and description of the finding are in Data Citation 1.

## Data Records

All Affymetrix GeneChip microarray raw data files and RMA normalized data were deposited as a Simple Omnibus Format in Text (SOFT) in the Gene Expression Omnibus (GEO) database^9,10^ with series accession number: GSE119135 (Data Citation 2). This includes the metadata and RMA normalized gene expression data for each sample. The SOFT file attributes and descriptions are found here: https://www.ncbi.nlm.nih.gov/geo/info/soft.html

## Technical Validation

The quality of the RNA extracted and purified from the samples is assessed by the *de facto* industry standard RIN obtained from the Agilent Bioanalyzer^7^. The validity of the gene expression data from the three studies was assessed using several technical validation approaches (Table 6) typically used for Affymetix gene expression arrays. The Bioconductor R packages “affyQCReport”, “simpleaffy” and “arrayQualityMetrics were used to generate quality control metrics and distributions of the data for visual inspection^11–14^. It can be a challenge or not possible to distinguish an outlier array from one which is different than others due to underlying biology. Hence, caution should be taken when evaluating the validity of microarray gene expression data in these studies due to perceived outliers since some samples may be responding to the time of exposure or the drug(s) may be targeting a given tissue preferentially. For brevity, we present only the array quality control (QC) metrics results of the Study 2 data.

### RNA integrity

The RIN numbers for the RNAs extracted from the samples in studies 1–3 were all ≥7 (Data Citation 1) indicating high quality, integrity and reliability for microarray analysis.

### Sources of Variability

To determine sources of variability which may constitute batch effects within a study, the log base 2 RMA normalized pixel intensity data were analyzed with the following analysis of variance (ANOVA) model:

$$Y_{ijklmn}=µ+A_{j}+B_{k}+T_{l}+D_{m}+S_{i}+\varepsilon_{ijklmn}$$

where *Y*_*ijklmn*_ represents the *n*^th^ observation on the *i*^th^ scan date (*S*) *j*^th^ drug given l^st^ (*A*), the *k*^th^ drug given 2^nd^ (*B*), the *l*^th^ tissue (*T*) and the *m*^th^ timepoint (*D*). *μ* is the grand mean for the whole study and the random *ε*_*ijklmn*_ are assumed to be normally and independently distributed with mean 0 and standard deviation *δ* for all measurements. Scan date is a random effect term in the model. As shown in Fig. 1b, according to the mean F statistics ratio, the major source of variability in Study 2 is attributed to the tissues.

### Cohesiveness of Samples

Given the major source of variability in Study 2 contributed by the tissues, we expect that the samples from the same tissues should be cohesive in 3-dimensional (3D) space when the log base 2 RMA data is analyzed by principal component analysis (PCA). When doing so, the 1^st^ three principal components (PCs) captures over 65% of the variability in the data (Fig. 1c). All but three of the Study 2 samples cluster by tissue: oxaliplatin followed by sunitinib in the kidney for 1 hr, D5W control followed by sorafenib in the liver for 24 h and D5W followed by sorafenib in the kidney for 24 h. Given that these samples are separated from their respective tissue samples and biological replicates, it is possible that the data from these arrays are outliers and should be considered with caution.

### Sample Quality

**Figure 2:**
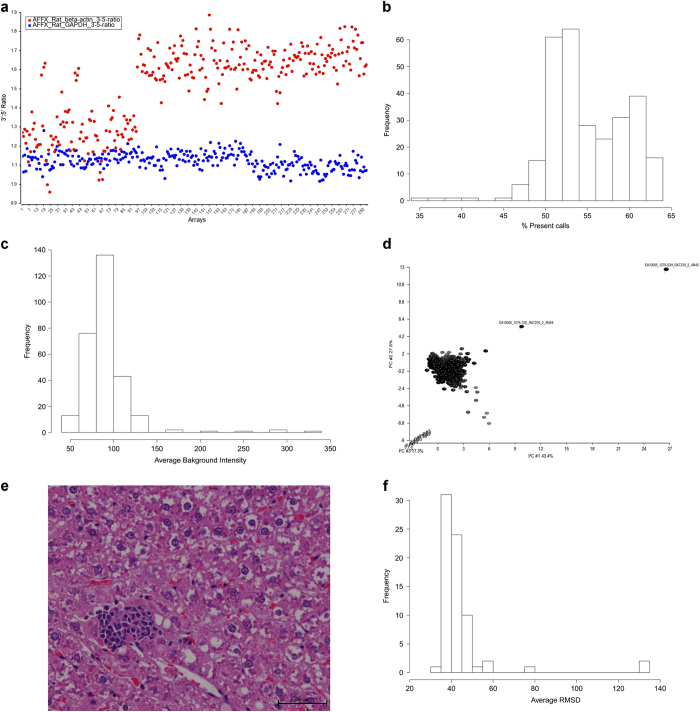
Data quality assessment. (**a**) 3′:5′ ratios of Study 2 samples. x-axis is the array index, y-axis is the 3′:5′ ratio of Actb (red) and Gapdh (blue). (**b**) Percent present calls for Study 2 arrays. (**c**) Average background intensity per Study 2 array. (**d**) PCA scatter plot of Studies 1, 2, and 3 samples using the RINs, percent present values, average background and the Actb and Gapdh 3′:5′ ratios. Two potential outliers are labeled. (**e**) Hematoxylin and eosin (H&E)-stained liver section from rat 4M48 in Study 1 group 4 administered temsirolimus (iv) followed by F68/PG/PEG400 (po) for 24 h magnified at 40x. The scale bar is 50 μm. (**f**) Distribution of the average RMSD per treatment group for Study 2.

The Affymetrix arrays contain internal control probes that target housekeeping genes beta-actin (*Actb*) and glyceraldehyde-3-Phosphate Dehydrogenase (*Gapdh*). The ratio of the 3′ probe and 5′ probe transcripts of *Actb* and *Gapdh* genes are indicators of sample quality and should typically not exceed 3 and 1.25 respectively. Ratios higher than these may indicate the presence of truncated transcripts or unsatisfactory RNA quality. The closer to 1.0, the better the RNA quality of the transcripts. As shown in Fig. 2a, the *Actb* and *Gapdh* 3′:5′ ratios for the Study 2 samples are within the limits. However, it is clear that the *Actb* 3′:5′ ratios for the kidney (array #s 97- 192) and liver samples (array #s 193 – 288) are much higher than most of them from the heart samples. The *Gapdh* 3′:5′ ratios are more consistent across all three tissues. *Actb* 3′:5′ ratios cap out at 1.9 and *Gapdh* 3′:5′ ratios have a mean of 1.52 with a standard deviation of 0.2.

### Array Characteristics

#### Percent present calls

The percent present calls are defined as the percentage of perfect match probes on the arrays that detect their targeted transcript as being present. As shown from the distribution of percent present calls for each array in Study 2, the majority have present call ≥50% (Fig. 2b). Twenty-six arrays have percent present calls <50; 23 from the livers samples, 2 heart samples and 1 from the kidney samples.

#### Background intensity

High background intensity reflects non-specific binding of labeled RNAs to the array. The distribution of the average background intensity within an array for Study 2 (Fig. 2c) reveals that 7 arrays have an average background pixel intensity ≥150; 6 from liver samples and 1 from heart.

### Outliers

PCA of the samples using the RINs, percent present values, average background and the *Actb* and *Gapdh* 3′:5′ ratios reveals two samples that are potential outliers (Fig. 2d): EA10065_1276-03H_RAT230_2_4M45 Temsirolimus (iv) followed by F68/PG/PEG400 (po) bone marrow treated for 6 h and EA10065_1276-10D_RAT230_2_4M48 Temsirolimus (iv) followed by F68/PG/PEG400 (po) liver treated for 24 h. However, the histology of the samples reveals that there are no differences in the bone marrow between all 4 rats in group 4 at 6 h or in the liver from rats in group 4 at 24 h. The bone marrow from all 4 biological replicates in the group was considered “normal” with no histologic findings. The minimal hematopoietic cell proliferation and minimal to mild cytoplasmic vacuolation seen in the liver (Fig. 2e) were observed in 3 of the 4 rats in group 4 at 24 h. These changes were not considered to be of biological significance and simply incidental background findings.

### Distance Between Biological Replicates

The gene expression distance between biological replicates is expected to be small. The average root mean squared distance (RMSD) is a measure of the average gene expression distance between biological replicates^15^. The average RMSD is essentially the mean Euclidean distance between all pairwise gene expression profiles (*x*_*i*_ and *x*_*j*_) with *n* number of probesets from *M* number of biological replicates:

$$\mathrm{average}\;\mathrm{RMSD}=\frac{\sum_{i<j}^{M}\left(\sqrt{\sum_{p=1}^{n}{\left(x_{pi}-x_{pj}\right)}^{2}}\right)}{N}\mathrm{for}\;i=1...M,\;j=1\ldots M.$$

In each study, there are *M* = 4 biological replicates per treatment (drug combination by time exposure) so *N* = *C*(4, 2) = M(M−1)/2 = 6 number of unordered combinations (4 choose 2). As shown in Fig. 2f, the majority of the treatments in Study 2 have an average RMSD <60. Replicate samples from 2 treatments in the kidney and 1 in the liver have average RMSD values >= 60:

1) D5W followed by Sorafenib in Kidney for 24 h average RMSD = 130.0

2) Oxaliplatin followed by Sunitinib in Kidney for 1 hr average RMSD = 76.7

3) D5W followed by Sorafenib in Liver for 24 h average RMSD = 130.2

### Array Pixel Intensity

**Figure 3:**
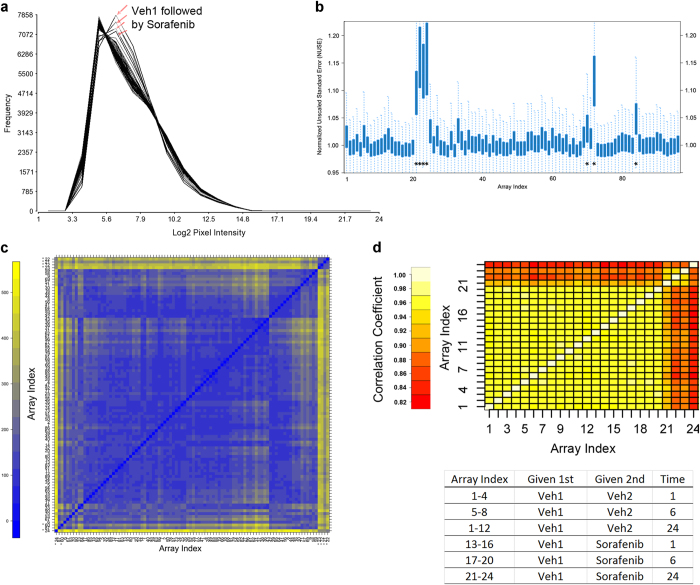
Comparison of samples. (**a**) Histograms of RMA normalized intensity data from Study 2 liver samples. (**b**) Boxplot distribution of NUSE values from Study 2 liver sample data. (**c**) Heat map of intensity data from Study 2 liver samples. (**d**) Array to array correlations for Study 2 liver samples treated with vehicle or sorafenib.

Typically, the distribution of the pixel intensity of the data from microarray arrays should be similar. However, there are cases where the distribution of one or more arrays are different than the other arrays. This could be a flag for questionable data. On the other hand, it can be the case that underlying biology from a treatment condition could be driving the differences in the distributional patterns of the data. Log transformation and normalization of the data help to make the data more normally distributed and comparable. As shown in Fig. 3a, the distribution of the RMA normalized, log base 2 pixel intensity data from the four biological replicates of the D5W followed by Sorafenib in Liver for 24 h treatment are different from the other arrays. Given that this treatment had a higher than normal average RMSD value and the distribution of the pixel intensity values from the biological replicates are different from the other arrays, it may be that this distribution pattern is more related to the treatment/biology than a systematic bias. However, further validation is warranted.

### NUSE

Another way to evaluate the distribution of the data is to assess the residuals from a probe-level error fitting model. Here, the probes from each array are compared to the probe-wise median of all the arrays to detect the difference. From the model, standard error (SE) from the probe expression and the median of all arrays for the probe are estimated to compute a normalized unscaled standard error^16^:

$$\mathrm{NUSE}({\hat{\theta }}_{ig})=\frac{SE({\hat{\theta }}_{ig})}{{\mathrm{med}}_{i}(\mathrm{SE}({\hat{\theta }}_{ig}))}$$

where ${\hat{\theta }}_{ig}$is the expression value for probeset *g* in array *i* and **med**() is the median. Plotting the NUSE values for each probe provides a standardized way of comparing the distributions of the data. The NUSE values should be close to 1. As shown in Fig. 3b for just the Study 2 liver samples, the four biological replicates for the D5W followed by Sorafenib in Liver for 24 h treatment (array indices 21-24) and three other arrays have higher NUSE value distributions than the other arrays. Again, since all four biological replicates for the D5W followed by Sorafenib in Liver for 24 h treatment behave the same, it is likely that this distribution pattern is related to the biology of the response. However, these samples and the other potential outliers require further quality assessment.

### Sample to Sample Correlations

One of the most intuitive ways of comparing array data is to visualize the pairwise correlation of the data from each sample. Heat maps of the intensity data from Study 2 liver samples (Fig. 3c) illustrate that the signal intensity from the D5W followed by Sorafenib in Liver for 24 h treatment have slightly higher pixel intensity data than the other arrays. Furthermore, the correlation matrix of the data from the samples (Fig. 3d) suggests that the replicates from the D5W followed by Sorafenib in Liver for 24 h treatment are highly similar to each other (correlation > 0.94) and very different from the vehicle controls (array indices 1-12) as well as the shorter time durations (1 hr and 6 h) of the exposure to the test article (arrays indices 13-20).

## Usage Notes

The gene expression data from the combination therapy can be used to assess the effect of a single drug on gene expression or the synergistic effect and order of administration of the drugs on gene expression. Assessing the difference of the effects of sunitinib or sorafenib on gene expression between tissues is possible provided that any potential batch effect due to the different studies is accounted for. Time dependency of the effects of the individual drugs or them in combination on gene expression within a tissue is capable by profiling the data across the durations of exposure.

## Additional information

**How to cite this article**: Davis, M. *et al*. Transcriptomic profiles of tissues from rats treated with anticancer drug combinations. *Sci. Data*. 6:180306 doi: 10.1038/sdata.2018.306 (2019).

**Publisher’s note**: Springer Nature remains neutral with regard to jurisdictional claims in published maps and institutional affiliations.

## Supplementary Material



## Figures and Tables

**Table 1:** Toxicogenomics studies to evaluate toxicity risk for combination therapy.

Study #	Main Drug	Combination Drugs	Tissues
1	Temsirolimus	Sunitinib Sorafenib	Bone Marrow
			Heart
			Liver
2	Oxaliplatin	Sunitinib Sorafenib	Kidney
			Heart
			Liver
3	Gemcitabine	Erlotinib Sorafenib	Skin
			Heart
			Liver

**Table 2:** Published gene expression studies in the rat and mouse in which the animals were exposed to the same chemicals as in this study.

GEO Accession	PubMedID	Publication Year	Species	Chemical	Combination Drug(s)/agents(s)	Platform
GSE37131	24047116	2013	Mouse	Temsirolimus	Bevacizumab	miRCURY LNA microRNA Array
GSE63902	25729387	2015	Rat	Oxaliplatin	Topotecan	Affymetrix Rat Genome 230 2.0 Array
GSE57811	25058030|26260164	2014|2016	Rat	Oxaliplatin	NA	Affymetrix Rat Genome 230 2.0 Array
GSE3210	16239200	2005	Rat	Oxaliplatin	NA	Codelink Rat Uniset 10 K
GSE60653	25909219	2015	Mouse	Oxaliplatin	Starvation	Affymetrix HT MG-430 PM Array
GSE51414	24264989	2013	Mouse	Oxaliplatin	Antibiotics	Affymetrix Mouse Gene 1.0 ST Array
GSE20147	20460542	2010	Mouse	Oxaliplatin	NA	Agilent-014868 Whole Mouse Genome Microarray 4 × 44 K
GSE47396	24147037	2013	Rat|Mouse	Gemcitabine	NA	miRCURY LNA microRNA Array
GSE57811	25058030|26260164	2014|2016	Rat	Erlotinib	NA	Affymetrix Rat Genome 230 2.0 Array
GSE57805	25058030	2014	Rat	Erlotinib	NA	Affymetrix Rat Genome 230 2.0 Array
GSE27641	24677197	2014	Rat|Mouse	Erlotinib	Diethylnitrosamine	Illumina expression beadchip
GSE98973	29051215	2017	Mouse	Erlotinib|Sorafenib|Sunitinib	NA	Illumina NextSeq 500
GSE27640	24677197	2014	Mouse	Erlotinib	Carbon tetrachloride	Illumina expression beadchip
GSE29415	22068661	2012	Mouse	Erlotinib	ras	NCI/ATC Mm-MEEBO
GSE6929	17935226	2007	Rat	Sunitinib	NA	Affymetrix Rat Genome 230 2.0 Array
GSE84048	28011623	2017	Mouse	Sunitinib	NA	Agilent-028005 SurePrint G3 Mouse GE 8 × 60 K Microarray
GSE50795	25017943	2014	Mouse	Sunitinib	NA	Agilent-014868 Whole Mouse Genome Microarray 4 × 44 K
GSE43053	25779766	2015	Rat	Sorafenib	NA	Affymetrix Rat Gene 1.0 ST Array
GSE8134	18303084	2008	Rat	Sorafenib	Hypoxia + VEGFR-2 inhibitor	Affymetrix Rat Genome 230 2.0 Array
GSE54857	24906623	2014	Mouse	Sorafenib	SV40 large T-antigen	Illumina expression beadchip

**Table 3:** Study 1 experimental design for temsirolimus combination with sunitinib or sorafenib.

Group	Treatment^∗^	Dose of Temsirolimus (mg/kg)	Dose of Sunitinib or Sorafenib (mg/kg)	No. of Animals per Time point						
				1 hour	6 h	24 h				
1	TEM vehicle (iv) followed by sterile water (po)	0	0	4	4	4
2	TEM vehicle (iv) followed by F68/PG/PEG400 (po)	0	0	4	4	4
3	Temsirolimus (iv) followed by sterile water (po)	0.3	0	4	4	4
4	Temsirolimus (iv) followed by F68/PG/PEG400 (po)	0.3	0	4	4	4
5	Temsirolimus (iv) followed by Sunitinib (po)	0.3	5	4	4	4
6	Temsirolimus (iv) followed by Sorafenib (po)	0.3	25	4	4	4
7	TEM vehicle (iv) followed by Sunitinib (po)	0	5	4	4	4
8	TEM vehicle (iv) followed by Sorafenib (po)	0	25	4	4	4
^∗^The interval between administration of the first and second agent was 30 min. F68 = Pluronic F68, PG = Propylene glycol, PEG 400 = Polyethylene glycol 400, PO = Per Os or Orally, IV = Intravenous						

**Table 4:** Study 2 experimental design for oxaliplatin combination with sunitinib or sorafenib.

Group	Treatment^∗^	Dose of Oxaliplatin (mg/kg)	Dose of Sunitinib or Sorafenib (mg/kg)	No. of Animals per Time point						
				1 h	6 h	24 h				
1	D5W (30 min infusion) followed by sterile water (po)	0	0	4	4	4
2	D5W (30 min infusion) followed by F68/PG/PEG400 (po)	0	0	4	4	4
3	Oxaliplatin (30 min infusion) followed by sterile water (po)	15	0	4	4	4
4	Oxaliplatin (30 min infusion) followed by F68/PG/PEG400 (po)	15	0	4	4	4
5	Oxaliplatin (30 min infusion) followed by Sunitinib (po)	15	5	4	4	4
6	Oxaliplatin (30 min infusion) followed by Sorafenib (po)	15	25	4	4	4
7	D5W (30 min infusion) followed by Sunitinib (po)	0	5	4	4	4
8	D5W (30 min infusion) followed by Sorafenib (po)	0	25	4	4	4
^∗^There was a 30-minute interval between the end of the first infusion dose and the administration of the second dose. D5W = 5% Dextrose in water, F68 = Pluronic F68, PG = Propylene glycol, PEG 400 = Polyethylene glycol 400, PO = Per Os or Orally, IV = Intravenous						

**Table 5:** Study 3 experimental design for gemcitabine combination with erlotinib or sorafenib.

Group	Treatment^∗^	Dose of Gemcitabine (mg/kg)	Dose of Erlotinib or Sorafenib (mg/kg)	No. of Animals per Time point						
				1 h	6 h	24 h				
1	0.9% saline (iv) followed by 0.5% CMC (po)	0	0	4	4	4
2	0.9% saline (iv) followed by F68/PG/PEG400 (po)	0	0	4	4	4
3	Gemcitabine (iv) followed by 0.5% CMC (po)	20	0	4	4	4
4	Gemcitabine (iv) followed by F68/PG/PEG400 (po)	20	0	4	4	4
5	Gemcitabine (iv) followed by Erlotinib (po)	20	150	4	4	4
6	Gemcitabine (iv) followed by Sorafenib (po)	20	25	4	4	4
7	0.9% saline (iv) followed by Erlotinib (po)	0	150	4	4	4
8	0.9% saline (iv) followed by Sorafenib (po)	0	25	4	4	4
^∗^The interval between administration of the first and second agent was 30 min. CMC = Carboxymethylcellulose, F68 = Pluronic F68, PG = Propylene glycol, PEG 400 = Polyethylene glycol 400, 10% Pluronic F68:propylene glycol:PEG 400 (15:42.5:42.5; w-w:w), PO = Per Os or Orally, IV = Intravenous						

**Table 6:** Technical validation approaches.

Validation Approach	Assessment
RIN	RNA integrity
ANOVA F ratio	Sources of variability and batch effects
PCA samples scatter	Cohesiveness of samples
beta-actin and GAPDH 3’:5’ ratios	Sample quality
% percent calls and average background intensity	Arrays with low expression detection and abnormal background intensity
Average RMSD	Average distance between biological replicates
Pixel intensity distribution	Distribution of the data from each array
NUSE	Precision (probeset homogeneity) of expression relative to other arrays
Heatmap and correlation matrix	Array to array similarity
RIN: RNA integrity number, ANOVA: analysis of variance, PCA: principal component analysis, GAPDH: Glyceraldehyde-3-Phosphate Dehydrogenase, RMSD: Root Mean Squared Distance, NUSE: normalized unscaled standard error.	
